# Purification and Characterization of a Thrombolytic Enzyme Produced by a New Strain of *Bacillus subtil*

**DOI:** 10.4014/jmb.2008.08010

**Published:** 2020-11-04

**Authors:** Jorge Frias, Duarte Toubarro, Alexandra Fraga, Claudia Botelho, José Teixeira, Jorge Pedrosa, Nelson Simões

**Affiliations:** 1 CBA – Biotechnology Centre of Azores, Faculty of Sciences and Technology, University of Azores, 9500-32 Ponta Delgada, Açores. Portugal; 2 CEB - Centre of Biological Engineering, University of Minho, 4710-057 Braga, Portugal; 3 CBMA – Centre of Molecular and Environmental Biology, University of Minho, 4710-057 Braga, Portugal; 4 INL - International Iberian Nanotechnology Laboratory, 715-330 Braga, Portugal; 5 ICVS - Life and Health Research Institute, University of Minho, 4710-07 Braga, Portugal

**Keywords:** *Bacillus subtilis*, subtilisin, fibrinolytic, thrombolytic, direct-acting thrombolytic enzyme

## Abstract

Fibrinolytic enzymes with a direct mechanism of action and safer properties are currently requested for thrombolytic therapy. This paper reports on a new enzyme capable of degrading blood clots directly without impairing blood coagulation. This enzyme is also non-cytotoxic and constitutes an alternative to other thrombolytic enzymes known to cause undesired side effects. Twenty-four *Bacillus* isolates were screened for production of fibrinolytic enzymes using a fibrin agar plate. Based on produced activity, isolate S127e was selected and identified as *B. subtilis* using the 16S rDNA gene sequence. This strain is of biotechnological interest for producing high fibrinolytic yield and consequently has potential in the industrial field. The purified fibrinolytic enzyme has a molecular mass of 27.3 kDa, a predicted pI of 6.6, and a maximal affinity for Ala-Ala-Pro-Phe. This enzyme was almost completely inhibited by chymostatin with optimal activity at 48°C and pH 7. Specific subtilisin features were found in the gene sequence, indicating that this enzyme belongs to the BPN group of the S8 subtilisin family and was assigned as AprE127. This subtilisin increased thromboplastin time by 3.7% (37.6 to 39 s) and prothrombin time by 3.2% (12.6 to 13 s), both within normal ranges. In a whole blood euglobulin assay, this enzyme did not impair coagulation but reduced lysis time significantly. Moreover, in an in vitro assay, AprE127 completely dissolved a thrombus of about 1 cc within 50 min and, in vivo, reduced a thrombus prompted in a rat tail by 11.4% in 24 h compared to non-treated animals.

## Introduction

Thrombolytic enzymes are currently used to treat cardiovascular diseases, the leading cause of death in the world. According to the World Health Organization [1], 17.5 million people died of cardiovascular diseases in 2012, and by 2030, around 23.3 million people are estimated to be afflicted by them. Therefore, the development of more effective and safer therapies is necessary. The three major cardiovascular diseases have thrombosis as a common factor [2], which results from the formation of a thrombus, a blood clot formed after an injury and composed mainly of fibrin. Under normal physiologic conditions, fibrin is hydrolysed by plasmin and the thrombus dissolved, but when fibrin is not hydrolysed due to various physiological reasons, the thrombus enters the bloodstream and increases the risk of thrombosis [3, 4]. Currently, the most common clinical treatment is the application of plasminogen activators, such as tissue plasminogen activators (t-PAs), urokinase (Uk), streptokinase (Sk) and nattokinase (Nk) [5-8]. These agents bind plasminogen to convert zymogen into plasmin (the active form in fibrin of the thrombus) and destroy undesired clots. However, bleeding complications caused by systemic activation of fibrinolytic mechanisms present a considerable risk associated with these enzymes [9, 10]. Therefore, many efforts have been made to develop more effective fibrinolytic agents that lack the known drawbacks of currently used plasminogen activators.

Microorganisms are a great source of fibrinolytic enzymes, and the enzymes used in most thrombolytic therapy (*i.e.*, Sk and Nk) have a bacterial origin. Streptokinase is a single-chain protein with 47 kDa, a pI of 4.7 and maximum activity between pH 7.3 and 7.6. It is produced by different strains of β-hemolytic *Streptococci*. Nattokinase is a single-chain protein with 20 kDa, a pI of 8.6 and is released by *Bacillus subtilis natto* [7, 11]. Thus, the search for new fibrinolytic enzymes is mostly concerned with microorganisms, which are relatively inexpensive, easy to implement with low cost and characterized by long lifetimes [12, 13].

In recent years, bacterial screenings resulted in the isolation and identification of a large number of productive fibrinolytic enzymes, such as *Bacillus thuringiensis* [14-16], *B. subtilis* [17], *Bacillus* sp. [18] and *Virgibacillus* sp.[19]. Special attention has also been given to microorganisms isolated from different fermented foods [20-22]. Despite fibrinolytic activity differences, none, to date, have identified amino-acid sequences or structural organizations able to explain the strong and weak fibrinolytic activities of different molecules [23-25]. Of the several studies on fibrinolytic enzymes, few have conducted in vitro and in vivo thrombolytic assays using a rat model [26-28]. Fibrinolytic enzymes should be first assessed in animal models to prove their in vivo activity and then evaluated for their potential in human clinical treatment. Thrombolytic agents currently approved by the FDA for patient use are plasminogen activators. Consequently, they do not have a direct-action mechanism over substrate fibrin. Fibrinolytic enzymes with direct-action mechanisms and safer properties are currently requested for thrombolytic therapy [29].

This work describes a new enzyme capable of degrading blood clots without impairing blood coagulation. This enzyme showed a direct-action mechanism by degrading the fibrin substrate without the need to activate other factors. It is also non-cytotoxic and constitutes an alternative to other thrombolytic enzymes (*e.g.*, Nk and Sk) known to cause hemorrhagic complications and other undesired side effects.

## Materials and Methods

### Screening for Fibrinolytic Activity

Twenty-four *Bacillus* sp. isolated from São Miguel Island in the Azores archipelago and belonging to our bacterial collection with caseinolytic activity, were tested for fibrinolytic activity. Isolates were plated in nutrient agar at 28°C for 24 h, and then half a colony of each isolate was transferred to plasminogen-rich and plasminogen-free fibrin agar plates to assess fibrinolytic activity. Plates were prepared by mixing 3 ml of fibrinogen solution (1.5% bovine fibrinogen (Sigma F4129, Portugal) in 20 mM Tris–HCl, pH 7.4), 100 μl of thrombin solution (Sigma T6884-100 U/ml) and 3 ml of 1% agarose in 5.5 cm Petri dishes and allowed to stand for 1 h at room temperature.

### Brute Extract and Enzyme Purification

The isolate S127e was grown in 250 ml of LB in a 1 L flask at 28°C, at 200 rpm for 18 h. The bacteria were separated by centrifugation at 10,000 g for 10 min, and the supernatant was filtered in a 0.22 μm cellulose acetate membrane (Millipore) and desalted by dialysis against Tris–HCl, 50 mM, and pH 8. Afterwards, the dialysate was concentrated by tangential flow filtration (TFF) using a polyethersulfone cassette with a cut-off of 10 kDa (Millipore) and stored at -80°C until used. For purification, 1 ml of 100× concentrated extract was applied in an anionic exchange column HiTrap-HQ (Amersham) coupled to an FPLC system (Amersham, Biosciences), equilibrated with 50 mM Tris-HCl, pH 8, and the protein was eluted in four steps of NaCl (0.1-, 0.2-, 0.5-, and 1 M). Active fractions were pooled, concentrated, and applied in a Superdex-75 column (Amersham), and protein eluted in phosphate buffer (PB), 50 mM, pH 6. Positive fractions were pooled and applied in a HiTrap-HS (Amersham), equilibrated with the same buffer, and eluted in a linear gradient of 0 to 1 M NaCl. Active fractions were pooled, desalted, concentrated and finally applied in a Mono S column, equilibrated in the same conditions as HiTrap-HS and eluted in a linear gradient of 0 to 1 M NaCl. Fractions were tested for fibrinolytic activity in fibrin agar plates and protein concentration determined using a NanoDrop (Thermo Scientific). The fraction with purified enzyme was desalted, concentrated and stored at -80°C until used.

### SDS–PAGE, Zymogram and MS/MS Analysis

Fractions eluted in the different chromatographies were suspended in non-reducing Laemmli buffer and analyzed in 12% SDS-PAGE using a Mini-Protean II Gel System (Bio-Rad). Proteins were stained with colloidal Coomassie. A zymogram was performed in 12% PAGE co-polymerized with 0.05% gelatine. After electrophoresis, the gels were washed twice for 30 min in 2.5% Triton X-100, followed by 30 min in deionized water and then incubated in 0.1 M Tris-HCl, pH 7.4, plus 0.1 M NaCl and 5 mM CaCl_2_, for 60 min at 37°C. Proteolysis was detected after Coomassie blue staining. The single protein band in SDS-PAGE resulting from the purified fraction was excised and prepared for MALDI-MS–MS analysis. Monoisotopic masses of the protein were obtained using a MALDI-TOF-MS model Voyager-DE-STR (Applied Biosystems). External mass calibration was performed using a mixture of peptide standards, PepMix1 (LaserBio Labs). The m/z masses were used to search in the NCBI non-redundant protein database (www.ncbi.nlm.nih.gov), using the public internet version of Mascot software (www.matrixscience.com) for identification. Trypsin digestion, a maximum of one missing cleavage, a cysteine carbamidomethylation and the possibility of methionine oxidation were selected as conditions in Mascot analyses.

### Optimal pH, Temperature and Ionic Concentration for Enzyme Activity

Determinations of optimal pH, temperature, and ions in buffers were performed in a microplate assay. Optimal pH was determined by the reaction of 10 μl of the purified fraction (0,50 μg/μl of protein) with 50 μl of 2%azoalbumin (w/v) in 40 μl of buffers at the desired pH (0.5 M acetate buffer for pH 4, 5 and 6; 0.5 M Tris–HCl for pH 7, 8, and 8.8) for 1 h at 37ºC. The reaction was stopped by the addition of 10 μl of trichloroacetic acid (100% w/v), vortexed, incubated on ice for 10 min and then centrifuged at 10,000 g for 5 min. The supernatant was transferred to a 96-well plate, neutralized with 10 μl of 0.1N NaOH, and the absorbance was measured at 450 nm in a microplate reader (Bio-Rad). The optimal temperature was determined at 16°C, 28°C, 37°C, 48°C, and 60°C, as described above in the buffer at pH 7. The effect of ions was investigated by adding CuSO_4_, ZnSO_4_, HgCl_2_, MnSO_4_, CaCl_2_, C°Cl_2_, MgSO_4_, and FeSO_4_ to the reaction mixture at a final concentration of 1 mM, performing the reaction at optimal pH and temperature.

### Substrate Specificity and Inhibitors

Proteolytic activity was measured using specific chromogenic substrates N-Suc-Ala-Ala-Pro-Met-pNA (Sigma M7771), Suc-Ala-Ala-Pro-Phe-pNA (Sigma S7388) and Suc-Gly-Gly-Phe-pNA (Sigma S1899) to analyze for chymotrypsin-like activity; Bz-Phe-Val-Arg-pNA (Sigma B7632) and Bz-Pro-Phe-Arg-pNA (Sigma B2133) for thrombin-like activity; Bz-Gly-Gly-Arg-pNA (Sigma C-7784) for urokinase-like activity; Z-d-Arg-Gly-Arg-pNA (Bachem 4029223.0100) for factor Xa like activity; and D-Ile-Pro-Arg-pNA (Sigma I0898) for plasminogen activator-like activity. For the enzymatic assay, 5 μl of 10 mM substrate was added to 45 μl of the enzyme, at a final concentration of 100 μg/ml diluted in 50 mM Tris–HCl, pH 7.4, and incubated at 37°C for 15 min. The formation of p-nitroaniline (pNA) was monitored at 405 nm every minute for 10 min in a microplate reader (Bio-Rad). One unit of enzymatic activity was defined as the amount of the enzyme that produced 1 nmol of p-nitroaniline per minute. The enzyme inhibition was tested using the Suc-Ala-Ala-Pro-Phe-pNA substrate in Tris–HCl 50 mM, pH 7.0, at 37°C, with the following protease inhibitors: phenanthroline, chymostatin, phosphoramidon, cystatin A, TPCK, PMSF, and antithrombin at a concentration of 100 μM; EDTA at a concentration of 10 mM; and leupeptin, E64, benzamidine, and soybean trypsin inhibitor at a concentration of 10 μM.

### Fibrinolytic and Fibrinogenolytic Activity Assay

Fibrinolytic activity was determined in a plasminogen-free fibrin plate, as described in section 2.1. After polymerization, 3 mm holes were punctured, filled with 10 μl of purified AprE127 or urokinase (Sigma U4010) with final concentrations of 5.30, 0.60, 0.07, and 0.01 mg/ml, and incubated at 37°C for 2 h. Images of the halos in the plate were taken at different times to measure the diameter of hydrolysis. The thrombolytic activity was calculated using the equation $\frac{R}{\surd t},$ where *R* is the radius of the halo, and *t* is the diffusion time [30]. The plasminogen activation assay was performed by reacting 71 μg purified S127e with 2.5 μg plasminogen (Sigma P7999) in fibrin plates and comparing with the same amount of urokinase. The fibrinogenolytic activity was assayed according to Salazar *et al*. (2007) with minor modifications [31]. Briefly, 5 μg of purified protein AprE127e was added to 50 μg of fibrinogen (Sigma F4129), suspended in 100 μl of Tris–HCl 50 mM, pH 7.4 and incubated at 37°C. Aliquots of 10 μl were taken from the reaction mixture at different time points (0, 5, 10, 20, 40, and 80 min), added to reducing Laemmli buffer, denatured at 95°C for 5 min and run in 12% SDS-PAGE.

### Effect on Euglobulin Clotting and Lysis

The effect of purified subtilisin S127e on blood coagulation and euglobulin clot lysis time was tested according to Smith *et al*. (2003) [32]. Human whole blood was collected at two regional health centers (Vila Franca and Nordeste), from adult volunteers after informed consent. The euglobulin fraction was prepared by adding 400 μl of citrated human plasma to 3.6 ml of 0.25% acetic acid (v/v), incubated on ice for 30 min and then centrifuged at 2,000 g for 10 min at 25°C. The supernatant was completely discharged, and the pellet was suspended in 400 μl of 100 mM sodium borate, pH 8.4, and vortexed for 1 min. The clot was then induced by adding 150 μl of euglobulin, 10 μl of thrombin and 30 μl of 0.1M Tris-HCl, pH 7.5, into a 96-well plate pre-warmed at 37°C. To assess the effect of the fibrinolytic enzyme on the clot lysis time, the buffer was replaced by purified AprE127. In the control (without clot formation), the thrombin was replaced by the buffer. Degradation products were measured at 405 nm every 5 min, for 720 min, using a microplate reader (Bio-Rad).

### Determination of APTT and PT

The effect of the purified AprE127 was tested on coagulation, on activated partial thromboplastin time (APTT) and prothrombin time (TT) using a coagulation analyzer (Diagnostica Stago). Each commercial reagent was reconstituted according to the manufacturer’s instructions. Then, 50 μl of human plasma was pre-incubated for 5 min at 37°C with 45.9 μg of the purified enzyme in 10 μl of 50 mM PBS, pH 7. As a control, non-treated human plasma was used. Coagulation time was measured in seconds until the full clot was formed.

### Thrombolytic Activity on Whole Blood Clots

To test the thrombolytic activity of the purified enzyme, whole blood clots were performed using fresh human blood. One piece of the blood clot with about 1 mm^3^ was placed in a well of a 96-well plate, washed three times with 50 mM PBS, pH 7, and incubated at 37°C for 5 min. To each well was added 80 μl of the enzyme, diluted in 50 mM PBS, pH 7, to a final concentration of 2.5 mg/ml. The lytic effect was followed and photographed every 10 min. Eighty microliters of urokinase at 2.5 mg/ml was used as the positive control, and 50 mM PBS, at pH 7, served as the negative control.

### Hemolytic Activity in Mammalian Red Blood Cells

Quantitative hemolysis was determined by plating 30 μl (75 μg/ml of purified enzyme) into 5% sheep blood agar plates (COS, Biomerieux) and incubated at 37°C. The halo resulting from blood lysis was checked after 4 and 18 h of incubation. Hemolytic activity was assayed using 5 μl heparinized whole human blood incubated with 95 μl (75 μg/ml of purified enzyme) in 50 mM PBS, pH 7.4, at 37°C for 45 min. Cells were then centrifuged at 2,500 *g* for 10 min, and the hemoglobin in the supernatant was determined at 545nm. Serum was used as a blank and 1%Triton X-100 in 50 mM Tris-HCl, pH 7.4, plus 150 mM NaCl as a positive control.

### Cytotoxic Assay

The cytotoxicity of the purified AprE127 was tested in Vero cells (Sigma-Aldrich No. 84113001) using the MTT assay. Then, 100 μl of Vero cells suspension, with about 105 cells, was applied per well in 96-well plates and incubated at 37°C for 24 h in a CO_2_ chamber. Afterwards, cells were treated with 2.37, 4.75, and 8.5 μg/ml of purified protein. Sodium azide, at 0.5 mM, was used as a positive control. After 18 h of incubation, 5 mg/ml of MTT solution was added to each well, and the formazan precipitate was dissolved in 100 μl of dimethyl sulfoxide after an incubation period of 4 h. The absorbance of the supernatant was read at 545 nm. Cell viability (%) was calculated as the OD in treatment / OD in the control × 100.

### In Vivo Assay for Thrombolysis

Six-week-old female Wistar rats weighing 180–200 g, supplied by Charles River in France, were kept in rooms with controlled temperature and humidity with a 12 h light/dark cycle. Animals were supplied with tap water and a normal pellet diet for at least three days before the experiments. Only animals with tails longer than 13 cm were used. The assay was performed according to Kamiya *et al*. (2010) [26]. The animals were anesthetized with dormitor (Medetomidine 0.5 mg/Kg) and Imalgene (Ketamine 75 mg/Kg). The anesthesia state was reverted with Atipamezole (1 mg/kg). Then, 200 μg of the purified enzyme in 500 μl of 50 mM PBS, at pH 7.5, was hypodermically injected per animal at ten different points along the tail of each rat (five points on each side). One hour later, the rats were intravenously injected in the tail vein with k-carrageenan suspended in the same buffer. The thrombus length was measured 24 and 48 h after the carrageenan injection. In the control group, animals were injected with only α-carrageenan in PBS.

### Gene Amplification and Sequencing

Chromosomal DNA of the isolate S127e was extracted using the Bacteria DNA Extraction Kit (Thermo) and used as the template to amplify 16S rDNA and S127e subtilisin genes. To identify the isolate, the amplification of the 16S rDNA gene was carried out using the forward primer 8F (5′-AGTTGATCCTGGCTCAG-3′) and reverse primer 1492R (5′-ACCTTGTTACGACTT-3′). The PCR amplification was carried out under the following conditions: 95°C for 3 min, followed by 35 cycles of 95°C for 30 sec, 46°C for 30 sec, 72°C for 1 min and, finally, 72°C for 2 min. The amplicons were purified using the PCR Clean-Up System (Promega) and then sequenced in STAB VIDA (Portugal).

The amplification of the S127e subtilisin encoding gene was performed with the degenerated primers Bk-5′-GCGCAATCCGTGCCTTACGGC-3′ and reverse Bk-5′-TTATTGTGCAGCCGCCTGTACGTTG-3′, designed based on the homologous references that were predicted by mass spectrometry analysis of the purified protein. The following PCR conditions were used: 95°C for 4 min, followed by 30 cycles of 95°C for 45 sec, 55°C for 45 sec, 72°C for 1.5 min and 72°C for 5 min. The PCR products were run in agarose gel. The amplified fragments were excised and then sequenced in STAB VIDA (Portugal).

### Bioinformatic Analysis

Protein motifs were identified using SMART (http://smart.embl-heidelberg.de/) and the conserved domain database from NCBI (http://www.ncbi.nlm.nih.gov/Structure/cdd/wrpsb.cgi). Sequence identities were determined by BLAST against the NCBI general database (http://www.ncbi.nlm.nih.gov/BLAST/). Multi-sequence analysis of homologous genes was performed using CLUSTAL W implemented in BioEdit 7.0. The 3D structure was predicted using the I-TASSER server (http://zhanglab.ccmb.med.umich.edu/I-TASSER).

### Sequences Submitted to Public Database

The 16S rDNA sequence of isolate S127e was submitted to NCBI, with accession number MN069579. The gene encoding the mature subtilisin, named AprE127, was submitted to NCBI, with accession number MF281668.

### Statistical Analysis

The statistical analyses were performed in Prism software (GraphPad Software, version 7.0, USA). All experiments were performed in triplicate and repeated three times with similar results. The data from in vivo thrombolytic assay were evaluated using Student’s *t*-test. Differences were considered statistically significant for *p* values < 0.10. The numbers of biological repetitions are depicted in the figure legends.

## Results

### Identification of Fibrinolytic Strain and Enzyme Purification

Six of the 24 isolates of *Bacillus* sp. demonstrated different rates of fibrinolytic activity. Based on the halo diameter of fibrin lysis, the isolate S127e, which presented the highest activity was selected. This isolate has a 16S rDNA (NCBI Acc. No. MN069579) and subtilisin named AprE127 (NCBI Acc. No. MF281668) sequences with 99.65% and 98.53% identity with those of *B. subtilis* P52 and J-5 strains, respectively. Therefore, it was assigned as a new isolate of *B. subtilis*. Cultures of *B. subtilis* S127e were deposited at the DSMZ (German Collection of Microorganisms and Cell Cultures) under the reference DSM 106532.

**Fig. 1:**
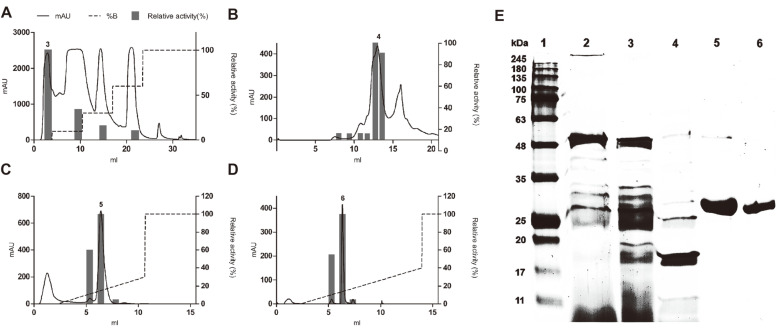
Purification of AprE127. The enzyme was purified in four consecutive chromatographies: anionic exchange (**A**), gel filtration (**B**) and cationic exchange (**C** and **D**); SDS-PAGE of proteins in the sequential steps of purification (**E**) [1 – Molecular markers; 2 – supernatant of bacterial culture; 3 – active fractions from anionic exchange; 4 – active fraction from gel filtration; 5 – active fraction from the 1st cationic exchange; 6 – active fraction from the 2nd cationic exchange].

**Fig. 2:**
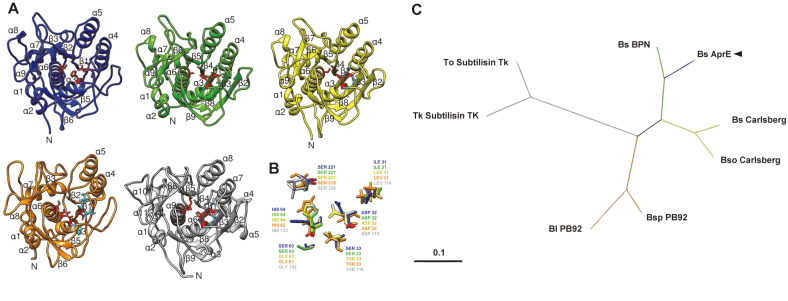
Predicted 3D structure of AprE127 and its comparison with close homologues (**A**): AprE127 in blue, Subtilisin BPN (1s01) in green, Subtilisin Carlsberg (1bfu) in yellow, Subtilisin PB92 (1ah2) in orange, and Subtilisin Tk (2z2x) in gray; Catalytic triad residues (red) and flanking non-conserved residues (cyan). Superimposed catalytic triad residues and flanking non-conserved residues (**B**): The amino acid residues are labeled and colored according to previously stated structures. The phylogenetic blast of AprE127 with the different groups of subtilisins (**C**): Bs_Carlsberg (1SBC_A); Bso_Carlsberg (WP_006636716); Bsp_PB92 (CAA01128); Bl_PB92 (AFK08970); Tk_Subtilisin_TK (2Z2Y_C); To_Subtilisin_TK (WP_088885546); Bs_BPN_natto (ACJ06132).

The fibrinolytic enzyme was purified using four sequential chromatographies (Figs. 1A-1D) with a purification factor of 78.2 (Table 1). The purified protein with fibrinolytic activity has an estimated molecular weight of 27 kDa based on SDS-PAGE and zymogram analyses (Fig. 1E). MALDI-MS-MS of the pure excised band originated six peptides (Table 2) covering 29% of the entire protein and matching a subtilisin-like serine protease of *B. subtilis* (NCBI Acc. No. AAY23643), with the significant score of 505 (scores greater than 75 are significant at *p* < 0.05). Using primers designed based on sequences identified by MS, an ORF was amplified with 825 bp that encodes for the purified enzyme. A BLAST search of the NCBI database showed that AprE127 had 0.98 identities with the subtilisins AprE176 (AHN52401) and DJ-4 (Acc. AAT45900), 0.97 with subtilisin K54 (AAC63365), and 0.85 with the subtilisins Nk1 (nattokinase, AAX35771) and Nat (nattokinase, AAC60424), all of them produced by *B. subtilis*. AprE127 encodes for an open reading frame of 272 amino acid residues, with a predicted molecular weight of 27.3 kDa, close to the molecular weight calculated by SDS-PAGE, and a predicted pI of 6.6. The molecular weight is close to the nattokinase [24, 34, 36], CK [35], subtilisin DJ-4 [38] and to the Subtilisin DFE [39], which are 27.7, 28.2, 29, and 28 kDa. However, it has a lower molecular weight than other proteases from *Bacillus* spp., like the jeotgal [42] and KDO-13 [43], which have 41 and 45 kDa, respectively. Also, at around 8.0, the isoelectric point resembled other fibrinolytic enzymes [38, 44] but was different from bpDJ-2, which is pI 3.5–3.7 [45]. SMART assembly analyses allowed the identification of a subtilisin-like domain between 25 to 253 aa, with the active site residues Asp-32, His-64, Ile-107, Leu-126, Asn-155, and Ser-221. It is known that almost all serine fibrinolytic enzymes belong to subtilisin of Bacillus origin and own the same catalytic triad with Asp-32, His-64, and Ser-221, without intramolecular disulfide bonds [24]. The order of catalytic residues Asp/His/Ser is a well-conserved feature indicating that AprE127 belongs to clan Bs and the S8 subtilisin family of serine proteases. In this triad, His takes part of the proton donor, and Asp is needed for the orientation of the imidazolium ring of His [33]. The 3D analysis of AprE127 is allowed to predict an identical fold to BPN subtilisin (PDB Acc. No. 1s01A) with a confidence score of 1.76 and an estimated model accuracy of 0.96 ± 0.05 (TM-score). This model evidenced the α/β-fold typical of the S8 subtilisin family, with a twisted central β-sheet containing seven strands surrounded by nine α-helices, with the active site residues converging to form the substrate binding loop (Fig. 2A), similar to other known crystal structures but with non-conserved amino acid residues flanking the catalytic triad (Fig. 2B).

The phylogenetic analyses showed that AprE127 is closer to the bacterial subtypes subtilisin BPN (S08.034) than to the subtilisins Carlsberg (S08.001), TK (S08,129), or PB92 (S08.003) (Fig. 2C).

### Biochemical Characterization of AprE127

AprE127 showed the highest activity in the specific substrate for subtilisins and chymotrypsins Suc-Ala-Ala-Pro-Phe-pNa, with 31.5 ± 1.4 U/min, followed by Suc-Ala-Ala-Pro-Met-pNa, with only 5.2 ± 0.6 U/min (Table 3). No activity was detected in substrates used to test enzymes involved in coagulation cascade (*e.g.*, thrombin and factor Xa), neither in substrates of plasminogen activators (*e.g.*, urokinase and plasminogen activator), all of them with Arg in the P1 position.

**Fig. 3:**
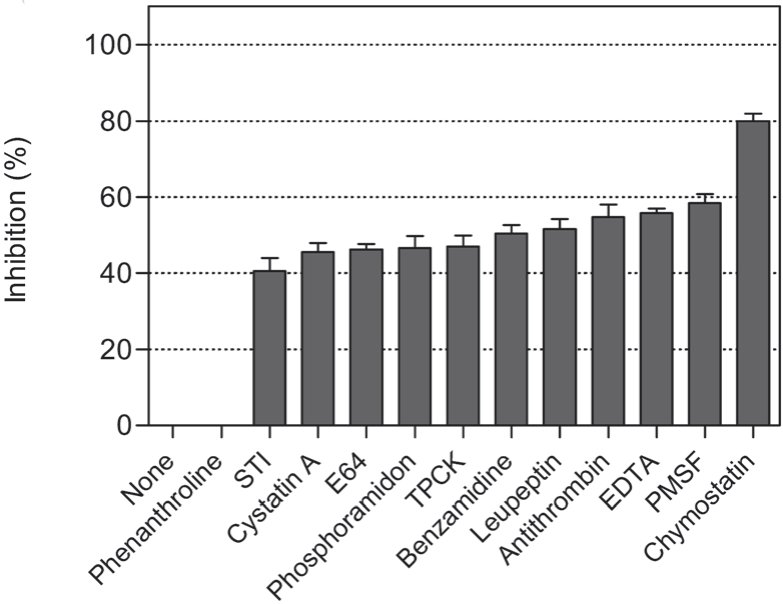
Biochemical characterization of AprE127 subtilisin. Effect of different inhibitors in the enzyme activity. Data are presented as mean ± SD, *n* = 3.

AprE127 activity was strongly inhibited by chymostatin (80%), a well-known inhibitor of serine chymotrypsin-like enzymes, and partially by PMSF (58 %), different from almost all other fibrinolytic enzymes (Fig. 3). AprE127 was active at pH within 6.0 and 8.8, with an optimum at 7.0, and in the thermal range 37–60°C, with an optimum at 48°C. The optimal temperature and pH were comparable to the optimal conditions of other relative serine proteases active at neutral and alkaline pH [37-39]. The catalytic activity of AprE127 increased by 80% in the presence of 1mM of Mg^2+^ (5.2 ± 0.6 U/min to 9.3 ± 0.5 U/min) and 10% in the presence of Ca^2+^, differently from other fibrinolytic enzymes like NAT, DC-4, and AprE176, which are activated by Ca^2+^ but not by Mg^2+^. On the other hand, AprE127 was inhibited in the presence of Cu^2+^ (80%), Zn^2+^ (80%), and Hg^2+^ (70%). It is well documented that divalent metal ions act as a cofactor in many BPN fibrinolytic enzymes, playing an important role in the catalytic reactions [13].

### Fibrinolytic and Thrombolytic Activity of AprE127

**Fig. 4:**
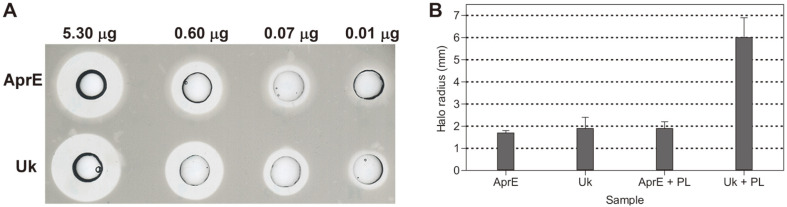
The activity of AprE127 subtilisin in fibrin and plasminogen activator assay. Comparison of fibrinolytic activities of AprE127 subtilisin (first line of wells) and human urokinase (second line of wells) at the same concentrations (**A**): 5.30, 0.60, 0.07, and 0.01 μg. Plasminogen activator assay (**B**): AprE127 subtilisin (AprE) and human urokinase (Uk) applied separately in the same concentrations, AprE127 subtilisin plus plasminogen (AprE + PL), human urokinase plus plasminogen (Ul + PL), both in the same concentration ratio. Data are presented as mean ± SD, *n* = 3.

The fibrinolytic activity of AprE127 and urokinase in a fibrin plate assay were compared, and both enzymes formed similar halos of fibrinolysis (Fig. 4A). Assays showed that both enzymes have identical constant activities, 1.85 and 2 for AprE and urokinase, respectively. Thus, the specific activity calculated for AprE was 3.8 U/ μg (urokinase unit equivalents). We also tested whether AprE127 was or was not an activator of plasminogen, by comparing its effect with that of urokinase. The halo formed in a fibrin plate by AprE127 alone did not differ from that formed by plasminogen plus AprE127, whereas a significant increase in the halo was observed with the application of plasminogen and urokinase (Fig. 4B).

**Fig. 5:**
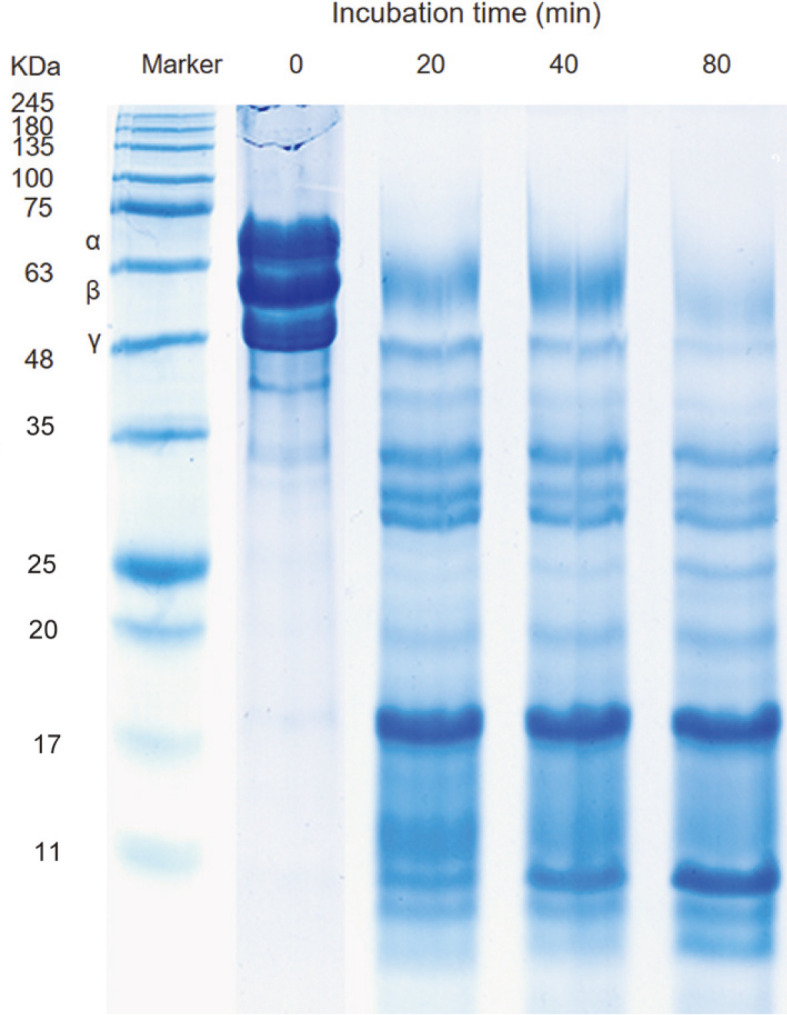
Digestion pattern of the AprE127 subtilisin in fibrinogen.

Moreover, the hydrolytic activity of AprE127 on fibrinogen was analyzed by an in vitro reaction. The SDS-PAGE profile of the digestion products of fibrinogen showed the complete hydrolysis of the α- chain in less than 20 min of incubation, whereas β- and γ- chains were not completely digested after 80 min of reaction (Fig. 5). This data points to the strong α- and moderate β- and γ-fibrinogenase activity of AprE127.

**Fig. 6:**
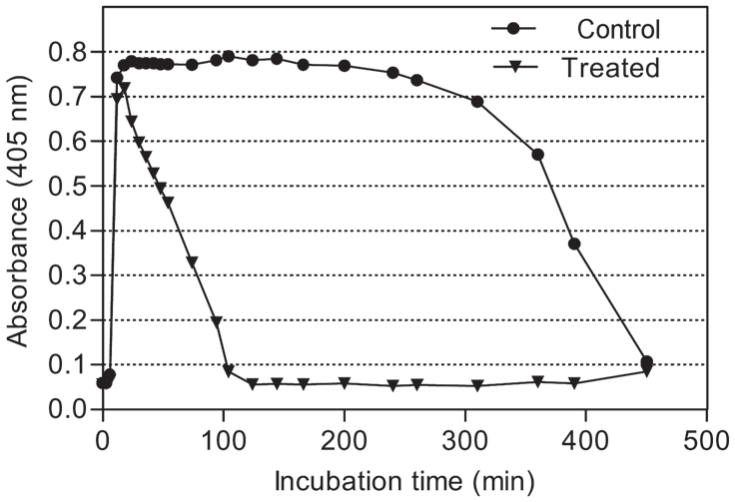
The activity of AprE127 subtilisin in fibrinolysis. Clot lysis time of euglobulin treated and untreated with AprE127 subtilisin. Data are presented as mean ± SD, *n* = 3.

The effect of AprE127 in coagulation was investigated using activated partial thromboplastin time (APTT), prothrombin time (PT) and euglobulin clot lysis time. Despite the strong activity of AprE127 in the hydrolysis of α-fibrinogen, a slight increase in APTT (from 37.6 to 39 s) and PT (from 12.6 to 13 s) was observed (Table 4), both within the normal range values (27–40 s for APTT and 11–14 s for PT). The assay performed with the blood fraction euglobulin that contained fibrinogen, plasminogen and PA allowed the confirmation that AprE127 did not impair the clot formation, which occurred within the same time and almost in the same extension in treated and non-treated euglobulin (Fig. 6). Nevertheless, AprE127 reduced the fibrinolysis time. In the control, the clot lysis curve was 462 min, whereas in euglobulin treated with AprE127, it was reduced to 100 min and started shortly after coagulation.

**Fig. 7:**
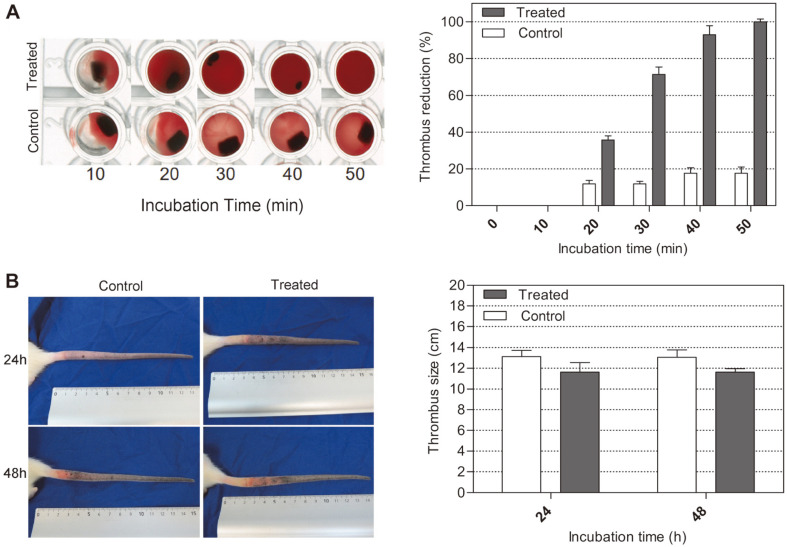
thrombolytic effects of AprE127 subtilisin. Lytic effect of the enzyme on whole blood clots performed in vitro (**A**); thrombolytic effect of the enzyme in an in vivo assay, on thrombus, induced by carrageenan in a rat tail (**B**). In both graphs, columns represent mean ± SD, *n* = 3. The asterisk shows the significant difference (*p* = 0.079) between the control and treated group (Student’s t-test).

The in vitro thrombolytic effect of AprE127 in thrombus with about 1 mm^3^ was tested. The treatment with 250 μg/ml of AprE127 completely dissolved the thrombus in about 50 min (Fig. 7A). The activity of AprE127 in degrading thrombi in vivo was assayed in the tails of Wistar rats in which thrombosis was induced by the injection of carrageenan, as previously reported [26, 27]. The average extension of the thrombi formed on the control animals was 13.13 ± 0.6 cm, while the extension of the thrombi formed in the animals injected with AprE127 was 11.63 ± 0.9 cm. This represented an 11.4% reduction in 24 h post-treatment (Fig. 7B). It should be noted that this effect of AprE127 remained 48 h post-treatment (Fig. 7B).

### Toxicity Assessment of the Enzyme

The hemolytic activity of AprE127 was tested in blood agar plates and whole blood. This enzyme did not cause any lytic halo in sheep blood cell plates after 18 h of incubation. In the whole blood hemolytic assay, 90% of hemocytes were viable after treatment with AprE127, whereas in the control group, only 5% of cells remained viable.

In Vero cells, the toxicity of AprE127 was under 5% after 18 h of incubation at concentrations up to 8.5 mg/ml, while cells treated with sodium azide (control) presented 80% mortality.

## Discussion

Due to medical interest in fibrinolytic enzymes produced by *B. subtilis* strains (*e.g.*, Nk), it was decided to screen for other potent thrombolytic enzymes as alternatives to currently approved plasminogen activators, as they lack direct-action mechanisms over substrate fibrin. The AprE127 encoding gene has high homology with the Nk gene of *B. subtilis* [34], the subtilisin BPN of *B. amyloliquefaciens* [35] and with the subtilisin E of Bacillus subtilis [13], but diverges from the TK, Carlsberg and Pb92 subtilisin groups. The superimposed structures of AprE127, subtilisin TK, Carlsberg, and Pb92 groups shows that some residues flanking the active site are not conserved (Figs. 2A-2B). For example, residue 31, which is flanking the active site residue Asp 32, is Ile in AprE127, but Leu in the other groups. Also, residue 33 in AprE127 is Ser, but Thr in the other groups. Another difference is at residue 63 flanking the His residue of the catalytic triad, where the polar amino acid Ser is switched to the less reactive amino acid Gly in the other three groups (Fig. 2B). These important findings are consistent with previous research hypothesizing that the catalytic activity can be influenced by small structural changes in the primary sequence flanking the active site residues. The substitution of amino acids flanking the conserved catalytic triad residues may contribute to forming different structural conformations that translate into different activities. The BPN group includes the largest number of fibrinolytic enzymes with the highest specificity to fibrin substrate of the entire subtilisin family [13]. Despite the great homology of these sequences, only a few of these fibrinolytic enzymes have high substrate specificity to fibrin, which can only be explained by evolutionary changes of the critical amino acid residues in the substrate-binding site [13]. Moreover, all the subtilisins belonging to AprE are fibrinolytic and have high substrate specificity to fibrin, differing from other proteases with fibrinolytic activity but with a broad spectrum [24,34,36-39].

Based on these results, AprE127 had a great affinity to substrates with large aromatic residues in the P1 position, similar to that of the homologous AprE176 [21] but different to the homologous fibrinolytic enzymes DC-4, NAT and BK-17, known to hydrolyse preferentially the polar residues lysine and arginine in the P1 position [39-41].

It should be noted that AprE127 was active under optimums of temperature, pH and Mg^2+^ concentration (1.2–1.4 mM in blood plasma) close to physiologic conditions.

AprE127 acted directly by degrading insoluble fibrin fibers; thus, it should be assigned as a plasmin-like protease. The specificity of this molecule against fibrin was demonstrated by amidolytic assays of different synthetic substrates. These data suggest that AprE127 directly degraded fibrin. It is worth noting that direct-acting thrombolytic agents, like plasmin, have a striking hemostatic safety advantage over plasminogen activators in animal models of thrombolysis and bleeding. In contradistinction to plasminogen activators, which risk bleeding at any effective thrombolytic dose, plasmin is tolerated without bleeding at several-fold higher amounts than those needed for thrombolysis. Plasmin has been safe in a current trial in patients with peripheral arterial or graft occlusion, and efforts are now directed towards the therapy of strokes caused by cerebral artery occlusion [29].

Moreover, despite the AprE127’s ability to quickly hydrolyse fibrin, it was also active in blood thrombus hydrolysis, as it was proved either by the reduction caused in fibrinolysis time of euglobulin or by the ability this enzyme had to dissolve thrombus in an in situ assay. Such observation suggested that this subtilisin is able to break down the rearrangements of fibrin in whole blood clots that are much more complex than in a simple fibrin plate agar. In blood clots, cross-linking bonds exist between the α chains and the γ chains of fibrin, thus promoting a clot markedly more resistant to proteolytic and mechanical disruption [46].

Along with the fibrinolytic effect, AprE127 was also able to cause fibrinogen degradation, a pattern revealing that α, β, and γ chains were cleaved at different degrees until fibrinogen was hydrolysed. Such a pattern (Aα > Bβ > γ) is similar to other fibrinolytic proteases [47-52]. Even though the pattern is similar, AprE127 completely degraded the three chains in comparatively less time than most of the subtilisins do. In contrast, AprE127 takes more time than the fibrinolytic metalloprotease purified from mycelia *Perenniporia fraxinea* [47] and than the fibrinolytic enzymes from *Paenibacillus polymyxa* EJS-3 [48], from *Pleurotus eryngii* [49] and the polychaete, *Neanthes japonica* [50]. However, the pattern of fibrinogen degradation by AprE127 is different from that of the fibrinolytic proteases of *Streptomyces* sp. CS684 and *B. subtilis* DC 33 [53, 54], which first hydrolyse β-chain.

In this fundamental work, it was proved that AprE127 was not toxic to cells in culture, and it is not hemolytic. Furthermore, this enzyme was able to cause a slight but evident reduction in the lengths of the infarcted region of a carrageenan-induced tail thrombosis test in rat in comparison with non-treated animals. This assay also suggested that the prophylactic effect of AprE127 injected at 0.4 mg.ml^-1^ resembled that described for Nk, which was administered at higher doses, between 1 or 2 mg.ml^-1^ [26]. Other studies, like Fujita *et al*. 1995 [28], have reported that nattokinase was four times more potent than plasmin in thrombus dissolution in rats, which is more potent than AprE127.

Despite that, contrary to AprE127, nattokinase not only degrades fibrin directly but also promotes the conversion of prourokinase to urokinase and increases t-PA formation [55], which is an indirect way of fibrin degradation by affecting plasminogen conversion to plasmin, and thus potentiates thrombolysis with less control of plasminogen activator levels in the blood, and, depending on the clinical case and the combination with other drugs, can lead to hemorrhagic complications [56].

In the case of AprE127, the mode of action is different, as it only acts directly by degrading insoluble fibrin fibers with activity similar to urokinase (Fig. 5A). Milner and Makise (2002) [57] have compared the fibrinolytic activity between nattokinase, urokinase and plasmin by the fibrin plate method, and nattokinase presented double the amount of fibrin lysis compared to the other two, while both urokinase and plasmin had the same fibrinolytic activity and thus supports the idea that AprE127, despite having lower thrombolytic activity than nattokinase, has the same fibrinolytic activity of plasmin, which is still considered a very potent thrombolytic agent.

In conclusion, our work demonstrates a new subtilisin, designated AprE127, that can be useful in overcoming important limitations of current agents, namely hemorrhagic complications. AprE127 was proved to be safe and to reduce thrombus extension in injected Wistar rats, with evident prevention of thrombus formation. Therefore, it has potential in the primary prevention of cardiovascular events. The uniqueness of AprE127 resides in a few differences in amino-acid sequences, which probably explain the differences observed in its substrate specificity and mode of action. More research should be done to completely elucidate the differences in AprE127 and to shed more light on the structure-function relationship.

## Figures and Tables

**Table 1:** Table of enzyme purification.

Steps	Total protein (mg)	Total act. (U)	Specf. act. (U/mg)	Purif. fold
Supernantant	145.6	1655	11	
HiTrap HQ	12.40	1604	129	11.7
HiTrap HS	1.68	1218	725	65.9
Mono HS	0.72	620	861	78.2

**Table 2:** Peptides identified in AprE127 subtilisin by Maldi – MS/MS.

Total score	Ms/Ms peptides	Calc. mass	Obsrv. mass	Error ± da	Ion score
505	SSLENTTTK	980.5	980.5	-0.0105	11
	LGDAFYYGK	1033.5	1033.5	-0.043	53
	HPNWTNTQVR	1252.6	1252.6	-0.0544	71
	AQSVPYGVSQIK	1276.7	1276.6	-0.0547	59
	APALHSQGFTGSNVK	1513.8	1513.7	-0.0542	136
	YPSVIAVGAVNSSNQR	1661.9	1661.9	-0.0712	163

**Table 3:** Comparative amidolytic activity in several synthetic substrates.

Synthetic substrate (1 mM)	Substrate hydrolysis (U/min)
N-Suc-Ala-Ala-Pro-Phe-pNA	31.5 ± 1.4
N-Suc-Ala-Ala-Pro-Met-pNA	5.2 ± 0.6
N-Suc-Gly-Gly-Phe-pNA	0
N-Bz-Phe-Val-Arg-pNA	0
N-Bz-Pro-Phe-Arg-pNA	0
N-Bz-Gly-Gly-Arg-pNA	0
Z-d-Arg-Gly-Arg-pNA	0
D-lle-Pro-Arg-pNA	0

**Table 4:** Activated partial thromboplastin time (APTT) and prothrombin time (PT) in whole blood sample treated and untreated with AprE127 subtilisin.

Assay	Control (s)	Treated (s)
APTT	37.6 ± 1.2	39 ± 1.5
PT	12.6 ± 0.4	13 ± 0.5
